# miRNAs Do Not Regulate Circadian Protein Synthesis in the Dinoflagellate *Lingulodinium polyedrum*

**DOI:** 10.1371/journal.pone.0168817

**Published:** 2017-01-19

**Authors:** Steve Dagenais-Bellefeuille, Mathieu Beauchemin, David Morse

**Affiliations:** Institut de Recherche en Biologie Végétale, Département de Sciences Biologiques, Université de Montréal, Montréal, Québec, Canada; University of Texas Southwestern Medical Center, UNITED STATES

## Abstract

Dinoflagellates have been shown to express miRNA by bioinformatics and RNA blot (Northern) analyses. However, it is not yet known if miRNAs are able to alter gene expression in this class of organisms. We have assessed the possibility that miRNA may mediate circadian regulation of gene expression in the dinoflagellate *Lingulodinium polyedrum* using the Luciferin Binding Protein (LBP) as a specific example. LBP is a good candidate for regulation by miRNA since mRNA levels are constant over the daily cycle while protein synthesis is restricted by the circadian clock to a period of several hours at the start of the night phase. The transcriptome contains a potential DICER and an ARGONAUTE, suggesting the machinery for generating miRNAs is present. Furthermore, a probe directed against an abundant *Symbiodinium* miRNA cross reacts on Northern blots. However, *L*. *polyedrum* has no small RNAs detectable by ethidium bromide staining, even though higher plant miRNAs run in parallel are readily observed. Illumina sequencing of small RNAs showed that the majority of reads did not have a match in the *L*. *polyedrum* transcriptome, and those that did were almost all sense strand mRNA fragments. A direct search for 18–26 nucleotide long RNAs capable of forming duplexes with a 2 base 3’ overhang detected 53 different potential miRNAs, none of which was able to target any of the known circadian regulated genes. Lastly, a microscopy-based test to assess synthesis of the naturally fluorescent LBP in single cells showed that neither double-stranded nor antisense *lbp* RNA introduced into cells by microparticle bombardment prior to the time of LBP synthesis were able to reduce the amount of LBP produced. Taken together, our results indicate that circadian control of protein synthesis in *L*. *polyedrum* is not mediated by miRNAs.

## Introduction

The circadian rhythm of bioluminescence in the marine dinoflagellate *Lingulodinium polyedrum* involves control over the synthesis of two proteins involved in the light emitting reaction, luciferase and luciferin binding protein (LBP) [1]. Both proteins are synthesized at the start of night phase and assembled into small organelles termed scintillons which then migrate toward the cell periphery [2]. The number of scintillons [3], as well as the cellular levels of both luciferase [4] and LBP [5] all correlate with the bioluminescence capacity of the cells. The regulation of both luciferase and LBP synthesis occurs at a translational level since mRNA levels for both are constant [5, 6]. The mechanism for this is still unclear, however, as there are conflicting reports concerning the action of an inhibitory protein binding to cis-acting sequences in *lbp* RNA during the day phase [7, 8]. No studies to date have examined a potential role for small regulatory RNA in regulating LBP synthesis.

In many different eukaryotes, including plants [9], animals [10] and diverse protists [11], double stranded RNA (dsRNA) has been shown to induce gene silencing. The mechanism may be related to a cellular defense against dsRNA viruses and involves degradation of the dsRNA into small 21–30 nucleotide fragments by an RNase III-like enzyme called Dicer [12]. The small RNA fragments, also known as small interfering RNA (siRNA), bind to Argonaute-Piwi (Ago-Piwi) family members and form RNA-induced silencing complexes (RISC) [13]. Proteins in the complexes are thought to cleave one of the two RNA strands [14], and the remaining strand on the RISC is used to target mRNA with a complementary sequence for degradation [15].

In addition to recognition of dsRNA, RISC-mediated gene silencing can also involve small regulatory RNA termed micro-RNA (miRNA). These miRNA are transcribed from the genome and also bind RISC, although their effect lies principally in an inhibition of protein synthesis [16]. The mechanism is conceptually similar to that described for siRNA, although in animals, most miRNA pair imperfectly with their targets and do not cleave their RNA targets [17], while in plants, translational inhibition by miRNA is genetically distinguishable from cleavage [18]. Thus, while these effects of miRNA on protein synthesis occur by a mechanism independent of RNA cleavage, inhibition of gene expression is the common endpoint of both types of RNAi-mediated mechanisms.

Several studies have now reported the presence of miRNAs in dinoflagellates. Small RNA sequencing of *Symbiodinium microadriaticum* revealed that 21 high quality miRNA candidates were present [19]. In *S*. *kawagutii*, 367 miRNAs were predicted from the genome sequence and 102 were identified by small RNA sequencing [20]. This latter study also reported that the levels of a miRNA predicted to target Hsp70 RNA decreased at higher temperatures, suggestive of a functional role in regulating HSP protein synthesis. Lastly, computational analysis suggested the presence of 18 miRNAs in the dinoflagellate *Alexandrium tamarense* [21].

In *L*. *polyedrum*, all the bioluminescence substrate (luciferin) is bound to LBP [22], which together with luciferase, constitutes the entire protein complement of the bioluminescent organelles termed scintillons [23]. Since luciferin is naturally fluorescent, scintillons can be readily observed in individual cells by fluorescence microscopy [24]. We tested if miRNAs were present in *L*. *polyedrum* using Northern blots and RNA sequencing. We also confirmed that both Dicer and Argonaute are found in the *L*. *polyedrum* transcriptome. Lastly, we tested if double stranded *lbp* RNA, introduced into cells using biolistics, would inhibit the nightly bout of LBP synthesis and thus block the synthesis of fluorescent scintillons. Our results show no evidence for inhibition of LBP synthesis by RNA interference.

## Materials and Methods

### Cell culture

*Lingulodinium polyedrum* cultures (strain CCMP1936; formerly *Gonyaulax polyedra*) were obtained from the Provasoli-Guillard National Center for Culture of Marine Phytoplankton (Booth Bay Harbor, Maine) and grown in f/2 medium [25] at a temperature of 19 ± 1°C under 12 hours light (50 μmol photons/m^2^/sec cool white fluorescent light) and 12 hours dark. ZT 0 (Zeitgeber Time 0) corresponds to the beginning of the subjective day (lights on) and ZT 12 to the onset of darkness.

### Plasmids and RNA synthesis

All *in vitro* transcription reactions were performed with a Riboprobe *in vitro* Transcription Systems kit (Promega Biotech) using 10 μg linearized plasmid DNA purified using a QIAprep kit (Qiagen). To prepare transcripts from a full-length LBP cDNA, a previously described 2.3 kb LBPa cDNA in pBluescript [26] was either linearized with *Bam*HI and transcribed using T7 polymerase to generate the antisense strand, or linearized with *Kpn*I and transcribed using T3 polymerase to generate the sense strand. To prepare smaller 1 kb transcripts, a 1080 bp *Pst*I fragment corresponding to the 5' end of the clone was first cloned into both SK and KS versions of pBluescript. The sense strand transcript was prepared using T7 RNA polymerase to transcribe a *Bam*HI digested SK vector, while antisense strands were synthesized using T7 to transcribe a *Hind*III digested KS vector. In addition, a 45 base oligoribonucleotide corresponding to the first 45 nucleotides of the LBPa and labeled with fluorescein at the 5’ end was synthesized in an antisense orientation (Sigma Genosys). Full length GFP transcripts in the sense orientation were obtained following linearization with *Spe*I and transcription using T7 polymerase of a pGEM-T Easy vector (Promega) into which a PCR insert of the mGFP6 sequence from the previously described pMDC107 vector [27] was ligated. All RNAs were purified by a chloroform extraction followed by ethanol precipitation. To generate double stranded RNA, equimolar quantities of sense and antisense strands were heated 15 minutes at 80°C in a heating block and the sample allowed to cool to room temperature over a two-hour period. All RNA samples were stored at -20°C until use.

### Biolistic bombardment

Bombardment was performed using sterile M-17 tungsten particles (1.1 μm mean diameter; Bio-Rad). A bead stock was prepared from 60 mg of microparticles that were vortexed vigorously for 5 min in 1 ml 70% ethanol, left standing for 15 minutes at room temperature, centrifuged at 2000xg for 2 sec, washed twice with DEPC treated H_2_O and finally resuspended in 1 ml 50% glycerol. For each experiment, 2–5 μg of purified RNA was added to a mixture of 40 μl of prepared beads, 40 μl 2.5 M CaCl_2_ and 16 μl 0.1 M spermidine (Sigma), and vortexed for 5 min. The microparticles were washed first with 70% ethanol, centrifuged, then washed again with 100% ethanol, centrifuged and finally resuspended in 20 μl 100% ethanol. Each bombardment used all of the final resuspended microparticles. This method is essentially similar to that used to introduce purified viral RNA into dinoflagellates [28] except for the use of tungsten rather than gold microparticles.

To prepare cells for bombardment, 50 ml of cell culture at roughly 10^4^ cells/mL was harvested by filtration on Whatman 541 paper either prior (LD12) or subsequent (LD18) to the daily bout of LBP synthesis. Care was taken to remove the filters when the cells had the appearance of a wet paste, as cells that were too dry did not survive the vacuum required for bombardment and cells that were too wet never contained microparticles after bombardment. Cells still on the filter paper were placed in a homemade particle delivery system 7–8 cm below a metal grid supporting the RNA coated beads. Air was pumped from the chamber, and when a vacuum 25 mm Hg was reached, a solenoid releasing a 5 msec pulse of 100 psi Helium was activated. After bombardment, cells were resuspended in 50 ml of fresh f/2 medium and returned to the culture room until observation the following night.

### Microscopic observations

Swimming cells were taken from night phase cell cultures exposed to culture room lights for thirty minutes prior to sampling and concentrated by a brief (15 sec) centrifugation at 13,000 x g. Exposure to light inhibits mechanical stimulation of bioluminescence which would otherwise reduce luciferin fluorescence [29]. With a thirty-minute exposure to light only 10–20% of the cells discharge their scintillons after mechanical stimulation, while almost all the scintillons discharge without the photoinhibition. Either a Zeiss Axio Imager (Jena, Germany) equipped with DIC and epifluorescence or a Zeiss LSM 510 confocal microscope was used for observations. Cells that contained tungsten microparticles were first identified using DIC microscopy. The fluorescence filters used with the Zeiss Axio Imager were Zeiss #34 filter set (ex 390/22 nm, em 460/50 nm) for scintillons and Zeiss #20 filter set (ex 546/12 nm, em 575–640 nm) for chloroplasts. For confocal microscopy, a 405 nm argon laser was used for excitation, and emitted light was passed through a beam splitter for simultaneous observation of scintillons (420–480 nm band pass filter) and chloroplasts (575 nm long pass filter). Relative luciferin fluorescence in individual cells was quantified using ImageJ by measuring mean pixel density within an area specific to each cells (delimited by an oval selection). All pictures to be compared were taken at the same time using the same settings on the microscope.

### Electrophoresis and Northern blotting

For the ethidium bromide staining of small RNAs, 10 μg samples of Trizol-extracted RNA from the higher plant *Solanum chacoense*, and from three dinoflagellates (*L*. *polyedrum*, *Pyrocystis lunula*, and *Amphidinium carterae*) were electrophoresed on 5% acrylamide gels. For Northern blots, 10 μg samples of RNA from the higher plant *Arabidopsis thaliana* and two dinoflagellates (*L*. *polyedrum*, and *S*. *kawagutii*) were transferred to a nylon membrane after electrophoresis on a 5% acrylamide gel and hybridized to an end-labeled radioactive probe prepared from the sequence 5’-acagattgcggcaaccgtgcag-3’ using the StarFire miRNA detection kit (IDT DNA). This probe corresponds to the most abundant *S*. *kawagutii* miRNA (scaffold 763_8250) previously shown to react on Northern blots with *S*. *kawagutii* RNA [20]. This miRNA sequence was found among our small RNA sequences (4588 reads) but does not match any sequence in our transcriptome.

### RNA sequencing and bioinformatics analysis

Samples of total RNA were isolated from *L*. *polyedrum* as described [30], and sent to Genome Quebec for sequencing on an Illumina HiSeq platform using the Illumina TruSeq miRNA library preparation with size selection protocol (McGill University and Genome Quebec Innovation Center, Montreal, Canada). The sequencing returned 159 million high quality reads, and the reads are directional since the primers ligated to the two ends of the RNA fragments are different. The raw data has been deposited in GenBank (Project number PRJNA69549, SRA accession number SRP007721). Fastq file manipulation was done using a local instance of the Galaxy bioinformatics suite [31]. FastQC was used to assess sequencing quality, cutadapt was used to remove the Illumina sequencing adaptors and polyA-containing reads, and bowtie2 [32] was used to map the reads against *L*. *polyedrum* rRNA sequences and *S*. *kawagutii* tRNA. Size selection of the remaining reads produced a set of reads ranging from 18–26 nucleotides. The collapse function in Galaxy was used to collapse these into a final set of 848,129 unique reads in order to determine the most abundant small RNAs. We removed from this collapsed read dataset all those containing fewer than 5 reads, leaving 133,861 collapsed reads. Among the 133,861 collapsed reads, 51,530 could be assembled to form 11,313 contigs with a 100% sequence identity over at least 14 bases. Of these, 948 had a length greater than the median length of 26 bases and contained only reads in the same direction. These 948 assemblies indicated the small RNAs contributing to them did not have the defined ends expected for *bone fide* miRNAs, so as with the miRPlex pipeline [33], they were also removed leaving a final dataset of 94,239 potential miRNA candidates. To test if any of the 94,239 sequences could form RNA duplexes with 2 base 3’ overhangs, the RNAs were first separated by length. All size classes were used one by one in a strand-specific BLAST search to detect all antisense partners with ≤ 3 mismatches and ≤1 unpaired bases (bulges). Only duplexes with 2 base 3’ overhangs were retained, and the results from all RNA lengths were then combined. These potential miRNAs were used to search for targets in the transcriptome using psRNATarget with an expected value of 2 or less [34]. To look for miRNAs in common with those in other dinoflagellates, we used MIRPIPE to search for all reported dinoflagellate miRNAs in the 4.9 million cleaned reads [35].

The domain structure of Dicers and Argoanutes was determined using the conserved domain function of the Blastp package [36]. For phylogenetic analysis of the Dicer proteins, five conserved domains were isolated from UNIPROT sequence annotation (including two RNAse III domains, the DEAD helicase domain, the dicer domain and the C terminal helicase domain) and aligned with the same domains taken from a variety of other Dicer sequences using Muscle. The five domain alignments were concatenated and submitted to the RAxML program at the CIPRES portal [37]. Reconstructions were visualized using Dendroscope [38]. Species include *Acyrthosiphon pisum*, *Agaricus bisporus*, *Ajellomyces capsulatus*, *Aspergillus niger*, *Caenorhabditis elegans*, *Cricetus griseus*, *Daphnia pulex*, *Glycine mas*, *Gorilla gorilla*, *Homo sapiens*, *L*. *polyedrum*, *Medicago trunculata*, *Mus musculus*, *Nannochloropsis gaditana*, *Oxytricha trifallax*, *Pan troglodytes*, *Paramecium tetraurellia*, *Penicillium digitatum*, *Peromyscus maniculatus*, *Phaseolus vulgaris*, *Physcomitrella patens*, *Phytophtora infestans*, *Pongo abelii*, *Populus trichocarpa*, *Rhodnius prolixis*, *Ricinus communis*, *Strigamia maritima*, *Tetrahymena thermophilia* and *Trichoplax adhaerens*.

## Results

**Fig 1:**
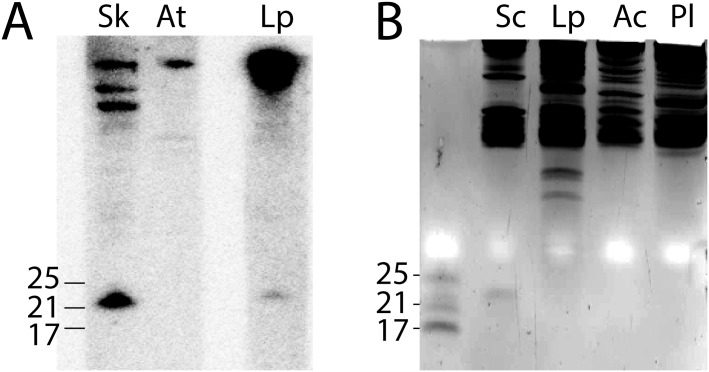
Gel electrophoretic analysis of miRNAs. (A) A 22 base miRNA highly expressed in *S*. *kawagutii* was tested by Northern blots on RNA extracted from the higher plant *Arabidopsis thaliana* and the dinoflagellates *S*. *kawagutii* and *L*. *polyedrum*. RNA samples were electrophoresed on acrylamide gels with ssDNA oligonucleotides of 17, 21 and 25 bases as size markers. (B) RNA extracted from the higher plant *Solanum chacoense* contains small RNA of 22 bases in length, whereas no RNA of this size class is detected in samples from the dinoflagellates *L*. *polyedrum*, *Amphidinium carterae* or *Pyrocystis lunula*.

The identification of miRNAs potentially able to regulate gene expression in *Symbiodinium* [19, 20] sparked our interest in the possibility that miRNAs might be involved in regulating gene expression in *L*. *polyedrum*, a species where the circadian clock regulates gene expression post-transcriptionally [6]. Northern blots using a highly expressed miRNA sequence from *S*. *kawagutii* as a sensitive test for miRNAs revealed *L*. *polyedrum* extracts contained a cross-reacting RNA of the correct size but of lower abundance (Fig 1A). However, ethidium bromide staining of small RNAs after gel electrophoresis indicated neither *L*. *polyedrum* nor two other dinoflagellate species showed detectable levels of RNAs of the correct size under conditions where miRNAs of higher plants are readily detected (Fig 1B). We conclude that while miRNAs do appear to be present in *L*. *polyedrum* they are found at lower levels than in higher plants.

**Fig 2:**
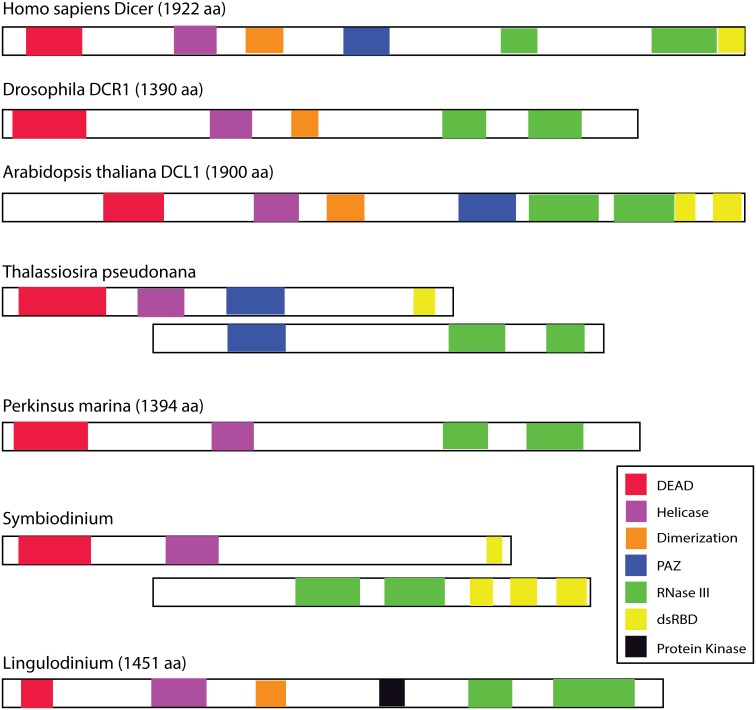
Schematic Dicer domain structures. The domain structures of animal, plant, diatom and dinoflagellate DICERs as determined by the conserved domain function in the blastp package.

Because of the apparent paucity of small RNAs in *L*. *polyedrum*, we next tested if the Argonaute and Dicer proteins required for miRNA synthesis were present in the transcriptome. We performed tBLASTn searches of our Velvet [30] and Trinity [39] assemblies using different domains selected from the Argonaute and Dicer sequences of *S*. *microadriaticum* [19]. Only one Dicer (GABP01078114) and one Argoanute (GABP01028167) sequence were recovered. The predicted *L*. *polyedrum* Dicer has a domain structure remarkably similar to that in plants and animals (Fig 2) as does the predicted Argonaute (S1 Fig). Phylogenetic analysis of five concatenated Dicer domains showed that while plant and animal sequences are clearly resolved, sequences from protists were found in a poorly supported clade along with fungal sequences (S2 Fig).

We next sought to confirm the presence of miRNAs using small RNA sequencing. Three samples, taken from cells at ZT2, ZT14 and ZT18, were prepared but only the latter contained sufficient RNA of the correct size for sequencing (150 bp in the size-enriched fraction, corresponding to the expected size of miRNA and adapters; S3 Fig). Illumina HiSeq using the ZT18 sample returned 159 M high quality reads. Of these, 8 M (5% of the initial reads) remained after removal of sequencing adaptors, rRNA, tRNA, polyA containing sequences and size selection (18–26 nucleotides) (Table 1). Reads corresponding to mitochondrial [40] or plastid sequences [41] were also removed, leaving 4,937,298 reads among which were found the probe used for Northern blots (4,588 reads). The size distribution of the reads shows peaks in small RNA abundance at 31 and 35 nucleotide lengths (S4 Fig) although a smaller peak is evident at 23–24 nucleotides. Of the 4.9 million reads, 678,838 (14%) had a match (BLASTn ≤ e-05) to sequences in the Trinity assembly (the probe used for Northern blots is not among these) suggesting they may be derived from mRNA. The majority of the small RNAs sequenced thus appears to be derived from non-coding RNA, which has been estimated to represent about 80% of the transcripts in human transcriptomes [42].

**Table 1:** Small RNA sequencing read counts.

Sample	Number of reads	%
High quality reads	159,181,068	100
rRNA removal	121,827,671	76.5
tRNA removal	118,629,442	74.5
polyA removal (>6 bases)	70,621,418	44.4
Size selection (18–26 nt)	8,599,813	5.4
Second rRNA and tRNA cleaning	8,038,881	5.1
Organelle RNA cleaning	4,937,298	3.1
Collapsed reads	848,129	
Collapsed reads with ≥ 5 copies	133,861	
Collapsed reads not in assemblies	94,239	

**Fig 3:**
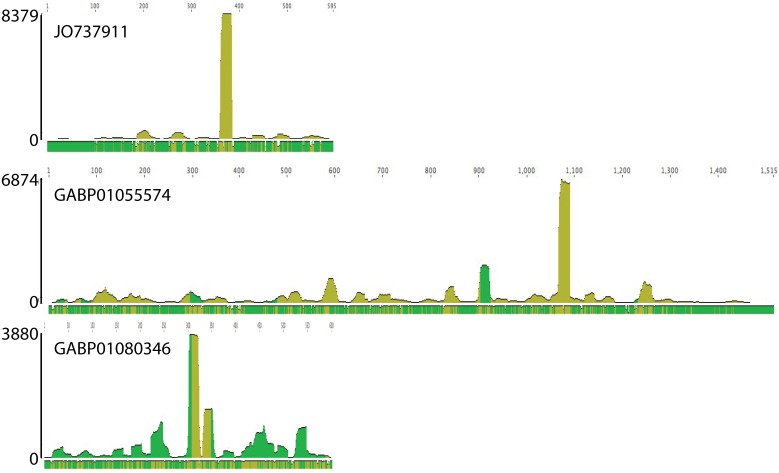
Patterns of read mapping to four different reference sequences. Twenty-eight Trinity sequences, corresponding to the 100 most abundant reads in the 4.9 M filtered sequence dataset, showed three patterns of assembled reads. The first (16 sequences) had reads lying all in the same direction (A). When the direction of the Trinity sequence encoding a protein was known, these reads correspond to the sense orientation. A second pattern contains very few sequences in the antisense direction, and these usually differ in sequence at their ends (B). A third pattern (C) had numerous antisense sequences, but these are distributed over the length of the transcript. The reference sequence in this case was a mitochondrial gene (cox1). All examples are all drawn to scale on the horizontal axis. The vertical axis shows the coverage (number of reads) at each base. Dark green indicates reads lie in both directions, while light green indicates all reads have the same orientation.

To further analyse the short read sequences with a match to the Trinity assembly, we first collapsed the 4.9 million read dataset and selected the top hundred in terms of read abundance. These top hundred most abundant reads corresponded to 28 different sequences in the Trinity database as identified by BLASTn searches (S1 Table). Each of these 28 Trinity sequences was used individually as a reference sequence to assemble the 4.9 M reads. A total of 652,369 reads were assembled to these 28 sequences, representing almost all (96%) of the reads with a match to the Trinity assembly. Remarkably, 16 of the 28 sequences contained only reads of the same direction (S1 Table; Fig 3). The libraries are stranded, so this result would be expected if all the sequences were derived from mRNA fragments, and a sense-strand direction was confirmed for those sequences corresponding to a known protein. Of the 12 sequences whose assembled reads were in both directions, 11 have very few reverse sequences—9 had reads in reverse direction whose sequences differed from the that in the assembly, and only two had sequences identical to that in the assembly. The last one had over 20% of the sequence in the reverse direction, but encoded a mitochondrial *cox1* sequence that had escaped filtering. This analysis supports the hypothesis that the small RNAs matching the Trinity assembly are likely mRNA fragments that were either resistant to digestion or were selectively amplified during preparation of the library for sequencing.

**Fig 4:**
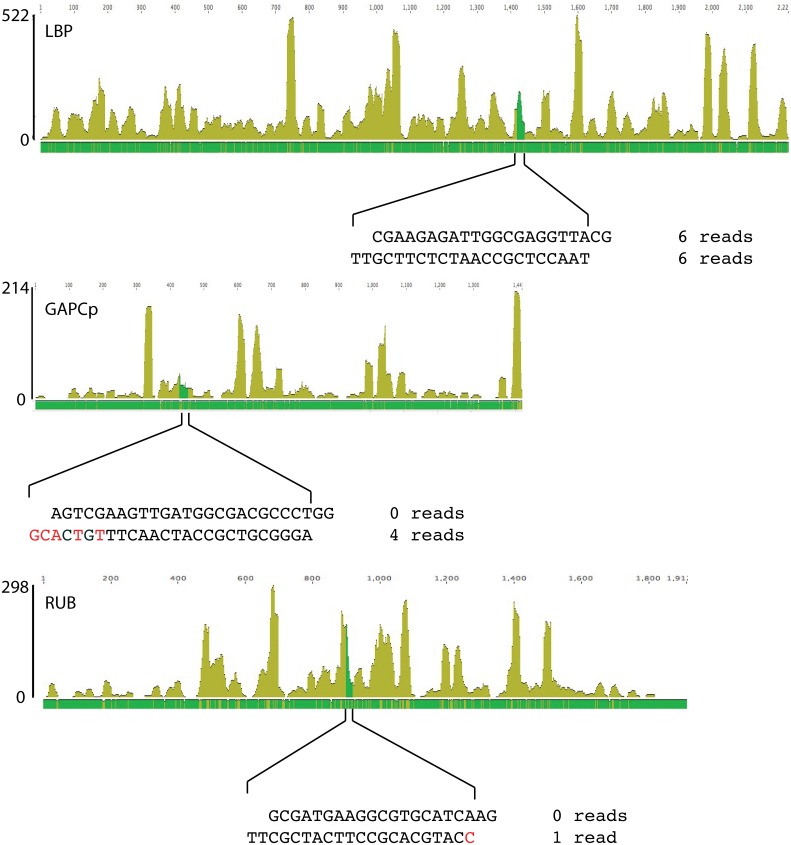
Patterns of read mapping to LBP, GAPCp or Rubisco as a reference sequence. (A) The majority of the reads mapped to LBP from the 4.9 million read dataset correspond to the sense strand. Six antisense sequences were found (dark green), and these potentially form duplexes with 3’ overhangs with six of the sense orientation reads as shown. (B) GAPCp. (C) Rubisco.

If miRNA were to be used as a general mechanism to mediate clock control over protein synthesis, then we might also expect to find miRNA sequences corresponding to the mRNA encoding circadian-regulated proteins. We thus assembled the 4.9 M reads to all known transcripts whose translation is under circadian control. With LBP as a reference sequence, 9,787 reads were recovered. Of these, six were in the antisense direction (Fig 4), and six of the sense strand reads could potentially produce a duplex RNA with two base pair overhangs at both 3’ ends with the antisense reads. This is far from the expected excess of miRNA over miRNA* as seen in *Symbiodinium* [20], suggesting these antisense reads are unlikely to be miRNAs. We also tested other known circadian regulated proteins, including the peridinin chlorophyll a protein (PCP) [43], a plastid-directed glyceraldehyde dehydrogenase (GAPCp) [44], Ribulose-1,5- bisphophosphate carboxylase/oxygenase (Rubisco) [45], superoxide dismutase (SOD) [46], oxygen evolving enhancer protein (OEE1) [47] and luciferase (LUC) [48]. No antisense sequences at all are associated with most of these (Table 2). The four antisense sequences mapping back to GAPCp are of an appropriate length (21 bases) to be miRNAs but differ in sequence at their last five bases (Fig 4) suggesting they may be derived from another RNA. Only a single antisense sequence is found associated with the Rubisco and there are no sense strand sequences able to form a duplex (Fig 4). None of the antisense small RNAs shown here are found in the 3’ UTR of the transcript.

**Table 2:** Small RNA read counts for mRNAs whose translation is circadian.

Protein	Total reads	Antisense reads
LBP	9787	6
LUC	5749	0
SOD	28	0
GAPCp	1571	4^a^
Rubisco	3731	1
PCP	6552	0
OEE1	439	0

^a^ These four 21 base sequences differ in their last 5 bases from GAPDH

**Fig 5:**
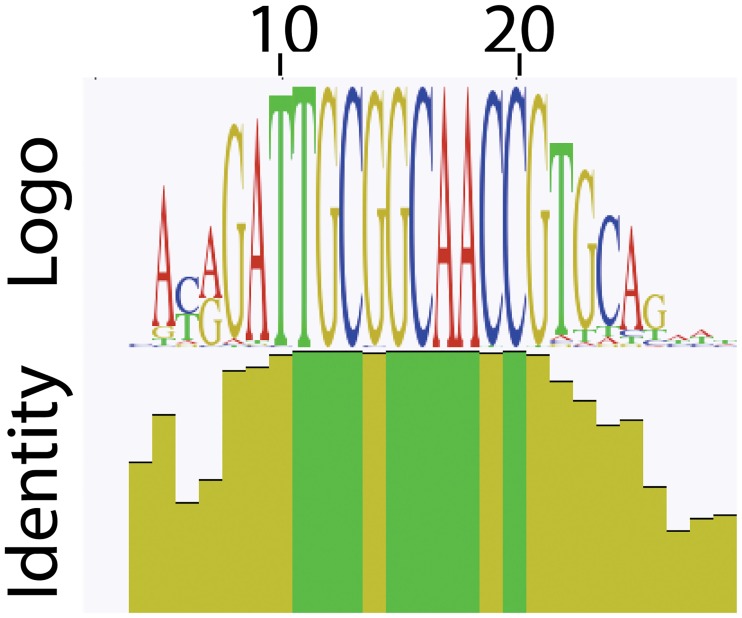
Sequence logo of sequences similar to *S*. *kawagutii* miRNAs. All but 27 reads from 25,195 with similarity to the 101 *S*. *kawagutii* miRNA could be aligned. Identical residues in the sequence logo correspond to the sequence of the probe used on Northern blots (5’-ACAGATTGCGGCAACCGTGCAG-3’).

We also tested if any of the 4.9 million reads were similar to miRNAs described for other dinoflagellates using both MIRPIPE and BLASTn searches. MIRPIPE indicated that most highly abundant miRNA (500 times greater than the next highest match) corresponded to the *S*. *kawagutii* miRNA used as a probe on our Northern blots. This was confirmed using BLASTn to match the 4.9 million reads with all the known *S*. *kawagutii* miRNA. This search recovered 25,195 matches, of which all but 27 could be aligned together. The consensus sequence, as seen in the sequence logo (Fig 5), is highly similar to that of the probe used on Northern blots in Fig 1.

**Fig 6:**
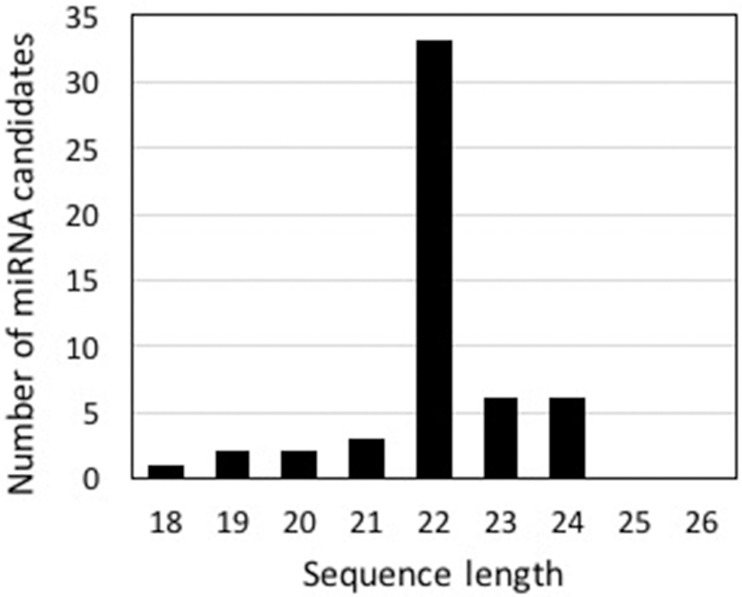
Length distribution of potential *L*. *polyedrum* miRNAs. The majority of the 53 unique small RNAs among the 94,239 collapsed sequences that are able to form duplexes with a 2 base 3’ overhang are 22 bases long.

As a more direct test for the presence of miRNA, we examined the 4.9 million reads for sequences able to form duplexes with 2 base 3’ overhangs. To facilitate this analysis, we first collapsed the 4.9 million reads to yield a dataset of 848,129 sequences and removed those present in less than 5 copies. We also removed sequences that could be assembled together to form longer contigs, mimicking what is done by the miRPlex pipeline [33]. The remaining 94,239 sequences contained 53 different small RNAs that were able to form duplexes with the 2 base 3’ overhangs expected for miRNAs, and most of these were 22 nucleotides long (Fig 6). We then tested if this group of potential miRNA candidates had potential targets in the transcriptome using psRNATarget. Using a stringent expect value of 0–2, psRNATarget identified 20 potential miRNA sequences with predicted hits to a total of 53 different sequences in the transcriptome. However, none of these potential target sequences is a known circadian regulated gene. We do note that when known (by perfoming BLASTx searches), the predicted miRNA is almost always in an antisense orientation with respect to the target (S2 Table).

**Fig 7:**
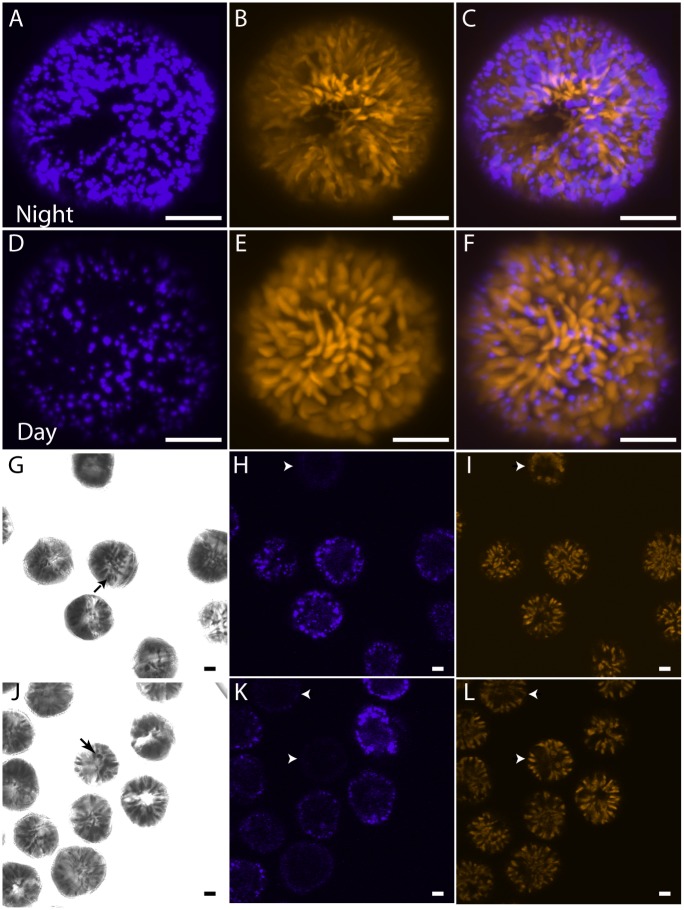
Fluorescence and bright field micrographs of *L*. *polyedrum*. A three dimensional reconstruction of a night phase (A-C) and a day phase (D-F) cell from a stack of confocal images shows scintillon fluorescence (A, D; Ex: 405 nm, Em: 420–480 nm), or for chlorophyll fluorescence (B, E; Ex: 405 nm, Em >575 nm) and a merged view of the two (C, F). The difference in scintillons numbers is under circadian control. Samples of cell cultures bombarded with tungsten microparticles show that small (~ 2 μm) microparticle aggregates (G, J) can be visualized inside cells (black arrows) by differential interference contrast (DIC) microscopy. The same fields examined for scintillon fluorescence (H, K) illustrates that cells with microparticles can have normal numbers of scintillons (H) or show the dark cell phenotype (K; white arrowheads); cells without microparticles can also show the dark phenotype (K). Chlorophyll fluorescence (I, L) in plastids serves as a marker for cell integrity. Scale bars are 10 μm.

**Fig 8:**
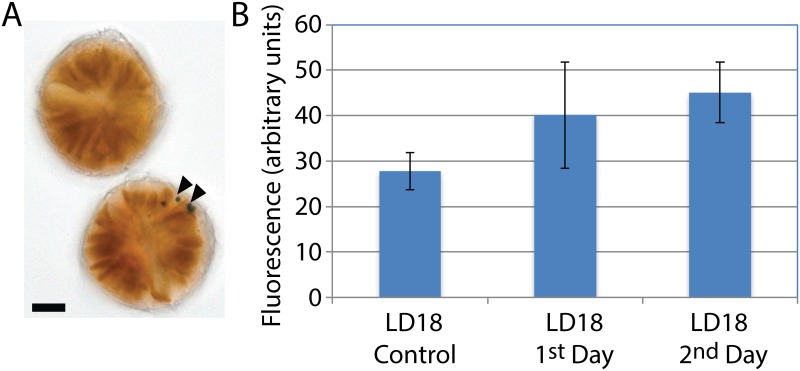
LBP RNAi does not significantly alter the luciferin fluorescence in cells. A) Cells with microparticles (arrow heads) under higher magnification (scale bar is 10 um). B) Total luciferin fluorescence in cells bombarded with microparticules lacking RNA at ZT12 and observed at ZT18, as well as cells bombarded with microparticules charged with *lbp* RNA at ZT12 and observed at ZT18 either on the same day (ZT18-1) or the following day (ZT18-2).

Despite the failure to find unambiguous examples of miRNA corresponding to circadian regulated genes, the presence of Dicer and Argonaute in the transcriptome prompted us to test the possibility that introduction of antisense or double stranded RNA could block protein synthesis. For this, we developed a microscopy-based assay for the amount of luciferin binding protein (LBP) in single cells. LBP binds the bioluminescence substrate luciferin with high affinity [22], and, since unbound luciferin is rapidly oxidized, the amount of luciferin fluorescence in unstimulated cells is proportional to the amount of protein. LBP synthesis is restricted by a circadian clock to a period of several hours just after the onset of the night phase [5], and is degraded at the end of the night phase resulting in a roughly ten-fold difference in luciferin fluorescence between day and night phase cells. Furthermore, since all the LBP is sequestered in bioluminescence organelles termed scintillons, the number of these organelles also varies ten-fold over the night (Fig 7) [3]. We predicted that blocking LBP synthesis in the cell by introducing double stranded LBP RNA prior to the bout of protein synthesis would result in night phase cells having levels of LBP fluorescence and a number of scintillons corresponding to what would normally be found in day phase cells. We thus bombarded cells at ZT12 using a gene gun firing microparticles either lacking RNA or charged with a 1000 bp double stranded *lbp* RNA. Cells were examined at ZT18 either the same day (LD18.1) or the following day (LD18.2). Cells that contain beads are readily visible with the microscope (Fig 8A), and when these cells were examined for luciferin fluorescence, no evidence was found for a decreased fluorescence in the cells bombarded with the *lbp* RNA (Fig 8B). This agrees with a visual estimate of the number of scintillons, so a visual estimate was also made for cells containing beads coated with antisense LBP RNA corresponding to 45 bases taken from the 3’UTR, or of 1000 bases antisense sequence corresponding to the 3’ region, or of 2000 bases of antisense sequence; none of the cells bombarded in these experiments showed day phase numbers of scintillons. We verified that RNA is adequately charged on the beads by bombarding tobacco pollen with beads charged with GFP RNA; GFP fluorescence is readily detectable in pollen tubes observed after germination *in vitro* (S5 Fig) as there is no background fluorescence in untransformed pollen (arrow) [49]. We did not observe GFP fluorescence in *L*. *polyedrum* using the same GFP RNA, and it is possible the high level of endogenous fluorescence in *L*. *polyedrum* hindered detection of low levels of GFP fluorescence. Alternatively, as we did not add the 22 nucleotide 5’ splice leader sequence characteristic of dinoflagellate mRNAs [50] to the GFP construct prior to RNA synthesis, it is also possible translation of the RNA may be impaired.

Curiously, while most cells containing microparticles had a normal night phase complement of brightly fluorescent scintillons, some cells had a dark phenotype apparently lacking scintillons (Fig 7H and 7K; white arrowheads). These dark cells are still alive, since they were taken from the swimming cells at the top of the culture flask and the distribution of red chlorophyll fluorescence into plastids appears normal (Fig 7I and 7L). However, cells without microparticles can also appear dark (Fig 7K, top arrow), so this phenomenon is more likely due to mechanical stimulation of the cell cultures which induces bioluminescence [51] and reduces luciferin fluorescence [24].

## Discussion

In the present study, we have evaluated the possibility that the dinoflagellate *L*. *polyedrum* uses RNA-mediated gene silencing to control gene expression, in particular to try and understand the circadian regulation of *in vivo* protein synthesis at a translational level. One experimental approach used small RNA sequencing to test for the presence of miRNAs. Out of three samples, only one contained sufficient small RNA for sequencing, and this sample returned 4.9 million filtered and size-selected reads that did not match to known organellem RNA, to rRNA or tRNAs. To search among these for miRNAs, we applied a single criterion—the ability to form duplexes with 2 base 3’ overhangs. In a final data set of 94,239 collapsed reads, from which we removed reads found in less than 5 copies and those able to assemble together into contigs, we found 53 small RNAs fulfilling this criterion. Twenty of these small RNAs had potential targets in the transcriptome as judged by psRNATarget (S2 Table), yet none of the potential targets represented a known circadian regulated gene. Interestingly, when the direction of the target genes in the transcriptome was determined using BLAST searches to the protein databanks, most of the potential miRNAs were found to be in an antisense orientation. Thus, while our data do not rule out the presence of miRNAs in *Lingulodinium*, they do rule out a role for miRNA in regulating the translation of known circadian-regulated transcripts.

We also examined reads assembling to the *lbp* RNA directly, as these had been removed from the duplex search described above because they assembled together into a long contig (Fig 4). Among the 4.9 million reads, 9,787 reads assembled to *lbp*. Among these, six antisense reads were found, and these potentially formed duplexes with two-nucleotide 3’ overhangs with six sense reads. However, it does not seem likely that the 6 antisense reads are miRNAs since the number of miRNAs should normally exceed the number of miRNA* [20]. In addition to LBP, reads mapping to other transcripts whose translation is regulated by the circadian clock are also primarily sense strand, ruling out potential regulatory small antisense RNA sequences as a general method mediating circadian clock control over translation (Table 2).

As a more direct test for RNA-mediated interference with LBP synthesis, we developed a test for evaluating if LBP synthesis could be inhibited *in vivo*. Normally, *L*. *polyedrum* cells display a transient spike in the synthesis rate of luciferase and LBP at the start of the night phase [5, 48] which results in a roughly ten-fold increase in the amount of both luciferase and LBP [4, 5] and an increase in the bioluminescence capacity of the cells. The newly synthesized LBP assembles, along with the reaction catalyst luciferase, into cytoplasmic bodies termed scintillons [2], and increased protein levels at the start of the night phase thus causes a ten-fold increase in the number of scintillons [3]. The regulatory mechanism has been reported to involve a trans-acting factor, found in day phase cells and able to bind a UG-rich region in the LBP 3' UTR [7], thus leading to the hypothesis that this factor might represent a circadian translational inhibitor. However, attempts to replicate the specific and circadian factor binding to this region were unsuccessful [8], leaving the mechanism of translational regulation open. One alternative, RNA-mediated gene silencing, is a wide-spread phenomenon that has been documented in plants, animals, and in various protists [11] including members of the Excavata (*Trypanosoma*), Chromalveolata (*Tetrahymena*), Archaeplastida (*Chlamydomonas*), Amoebozoa (*Dictyostelium*) and Opisthokonts (*Neurospora*). Furthermore, miRNA complementary to RNA encoding heat shock protein has a decreased abundance in heat-shocked *S*. *kawagutii* [20], consistent with a role in regulating protein synthesis. RNAi-mediated gene silencing thus represents a potential mechanism for mediating clock control over LBP synthesis that should be tested by experiment.

We tested the ability of several different antisense or double stranded *lbp* RNA preparations to block the normal circadian increase in scintillon numbers that occurs during the night phase of the dinoflagellate *Lingulodinium* as a result of LBP synthesis [3]. We predicted that night phase cells in which LBP synthesis had been blocked would have only day phase numbers of scintillons. However, in observations of over a hundred swimming cells that contained visible microparticles by microscopic evaluation, no cells with day phase numbers of scintillons were found. Instead, the only phenotype observed was the presence of "dark" cells, apparently lacking scintillons. We ascribe this dark phenotype to a lack of the bioluminescence substrate, as luciferin fluorescence in the scintillons is reduced by bioluminescence [24]. Since mechanical agitation of the cells results in bioluminescence, the dark cells likely arose because of bioluminescence emission occurring during the centrifugation of cells for microscopic observations. We conclude from this that RNAi-mediated gene silencing is unable to account for the circadian rhythm in LBP synthesis.

In these studies, RNA has been introduced into dinoflagellates by microparticle bombardment, a technique known to induce gene silencing in both plants [52] and animals [53]. Furthermore, RNA purified from HcRNAV, a single stranded RNA genome virus infecting *Heterocapsa circularisquama* [54] is functional after bombardment of cells as assessed by replication of viral RNA and synthesis of viral like particles in the cytoplasm by transmission electron microscopy [28]. This suggests that bombardment with RNA-coated microparticles can be used to test involvement of miRNAs in circadian control of translation if gene expression can be measured on a cell-by-cell basis. While experiments of this nature have not yet been reported for dinoflagellates, introduction of heterologous RNA may be useful for study of gene expression, as despite other reports of dinoflagellate transformation [55, 56], we have so far been unable to express reporter genes in *L*. *polyedrum*.

Is it possible that the scintillons in *L*. *polyedrum* can form without LBP? Some bioluminescent dinoflagellates, such as *Pyrocystis fusiformis*, do contain scintillons [57] yet have no detectable LBP activity [58]. However, while these structures clearly form in the absence of LBP, their composition is unknown, so it is difficult to determine if they could serve as a model for a *L*. *polyedrum* scintillons lacking LBP. In contrast, scintillons from *L*. *polyedrum* have been purified and characterized. These contain only luciferase and LBP in roughly equimolar amounts [23], and it seems unlikely that they could still form if one of their two protein constituents was absent. More importantly, the fluorescent substrate luciferin binds to LBP [22], and even if scintillons could form without this protein, they would not be visible by fluorescence microscopy as the luciferin would not be sequestered.

It is noteworthy that in the protists where RNA-mediated gene silencing has been demonstrated, the genome sequence has now been shown to encode Dicer-like, Argonaute-Piwi and RdRP proteins [11]. Furthermore, miRNA has now been characterized from the unicellular green alga *Chlamydomonas* [59, 60]. The broad taxonomic range of species containing components used for RNA-mediated gene silencing suggests this mechanism may have evolved prior to the divergence of the different eukaryotic lineages. However, among the Alveolates (encompassing Dinoflagellates and Apicomplexans), no homologs to Dicer, Ago or RdRP are found in the sequenced genomes of the apicomplexans *Plasmodium* and *Cryptosporidium* [11, 61, 62]. Furthermore, no definitive demonstration of RNAi mediated gene silencing has appeared for *Plasmodium* and *Cryptosporidium*. However, the apicomplexan *Toxoplasma* expresses a truncated Ago that has been implicated in an effect on gene expression [63], so that RNA-mediated gene silencing may not necessarily have been lost in all Alveolates. DICER and AGO have been reported in *S*. *microadriaticum* [19] although no functional assessments of miRNA function were performed. However, it is clear that RNAi does not significantly modulate *lbp* gene expression in *L*. *polyedrum*.

## Supporting Information

S1 Fig Domain structure of Argonaute proteins. The domain structures of animal, plant, diatom and protist Argonautes as determined by the conserved domain function in the blastp package. Conserved domains in the dinoflagellate sequences include PAZ (Piwi-Argonaute-Zwille), Argonaute N terminal (ArgoN) and PIWI PIWI-like. Animal sequences also contain an Argoanute-like (ArgoL) and a GAGE domain of unknown function.
(PNG)

S2 Fig The position of the *L*. *polyedrum* Dicer in phylogenetic reconstructions. Five Dicer domains from a range of animal plant fungal and protist sequences were aligned and concatenated. RAxML was used to generate the phylogeny from the concatenated alignment. Sequence names correspond to the first five letter of the genus as provided in the methods; sequences were numbered when more than one example was recovered.
(TIF)

S3 Fig Agilent DNA chip analysis of RNA samples. Three different samples, taken from three different times, were processed by adapter addition and assessed by electrophoresis. The expected size of miRNAs after adapter ligation is ~150 nucleotides.
(DOCX)

S4 Fig Length distribution of the sequenced small RNAs. The number of sequences is reported as a function of the sequence length for the 4.9 million small RNAs sequenced.
(PNG)

S5 Fig Pollen grains bombarded with microparticles charged with *gfp* RNA express GFP after germination. Untransformed pollen has no background GFP fluorescence (arrow).
(PNG)

S1 Table The hundred most abundant read sequences map to 28 different transcripts. (PDF)

S2 Table Predicted miRNA targets. (PDF)
