# Supplementary figures and images for: miRNAs Do Not Regulate Circadian Protein Synthesis in the Dinoflagellate Lingulodinium polyedrum
Source: PLoS One. 2017 Jan 19;12(1):e0168817. doi: 10.1371/journal.pone.0168817 (PMC5245829; doi:10.1371/journal.pone.0168817)

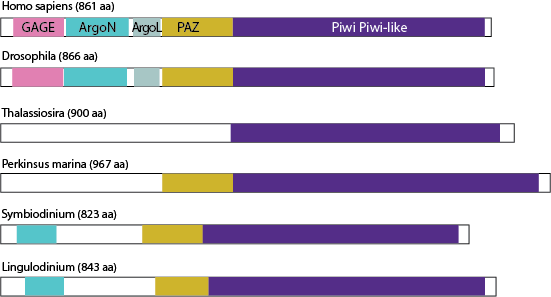

Supplement: S1 Fig — The domain structures of animal, plant, diatom and protist Argonautes as determined by the conserved domain function in the blastp package. Conserved domains in the dinoflagellate sequences include PAZ (Piwi-Argonaute-Zwille), Argonaute N terminal (ArgoN) and PIWI PIWI-like. Animal sequences also contain an Argoanute-like (ArgoL) and a GAGE domain of unknown function. (PNG) [file pone.0168817.s001.png]

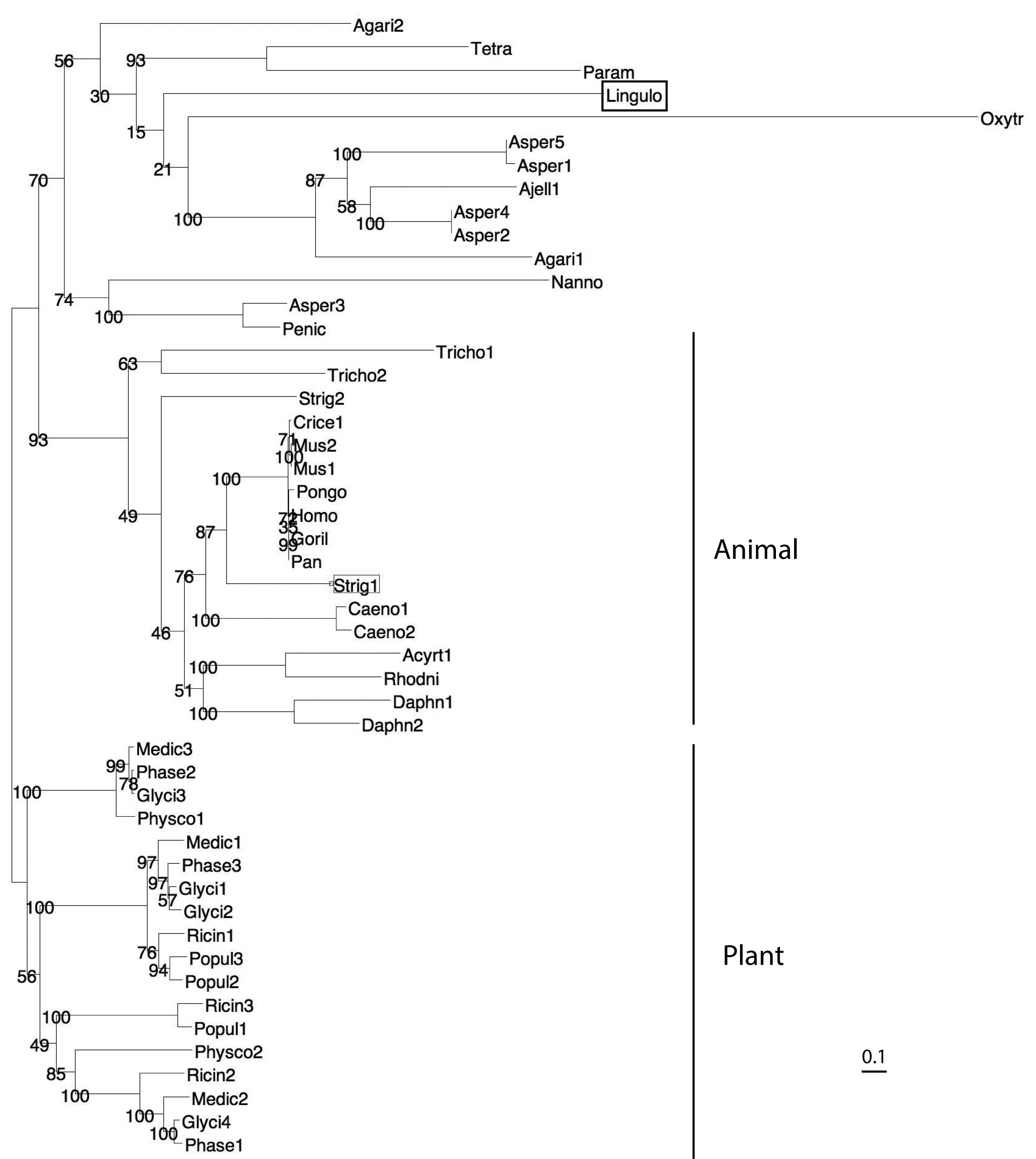

Supplement: S2 Fig — Five Dicer domains from a range of animal plant fungal and protist sequences were aligned and concatenated. RAxML was used to generate the phylogeny from the concatenated alignment. Sequence names correspond to the first five letter of the genus as provided in the methods; sequences were numbered when more than one example was recovered. (TIF) [file pone.0168817.s002.tif]

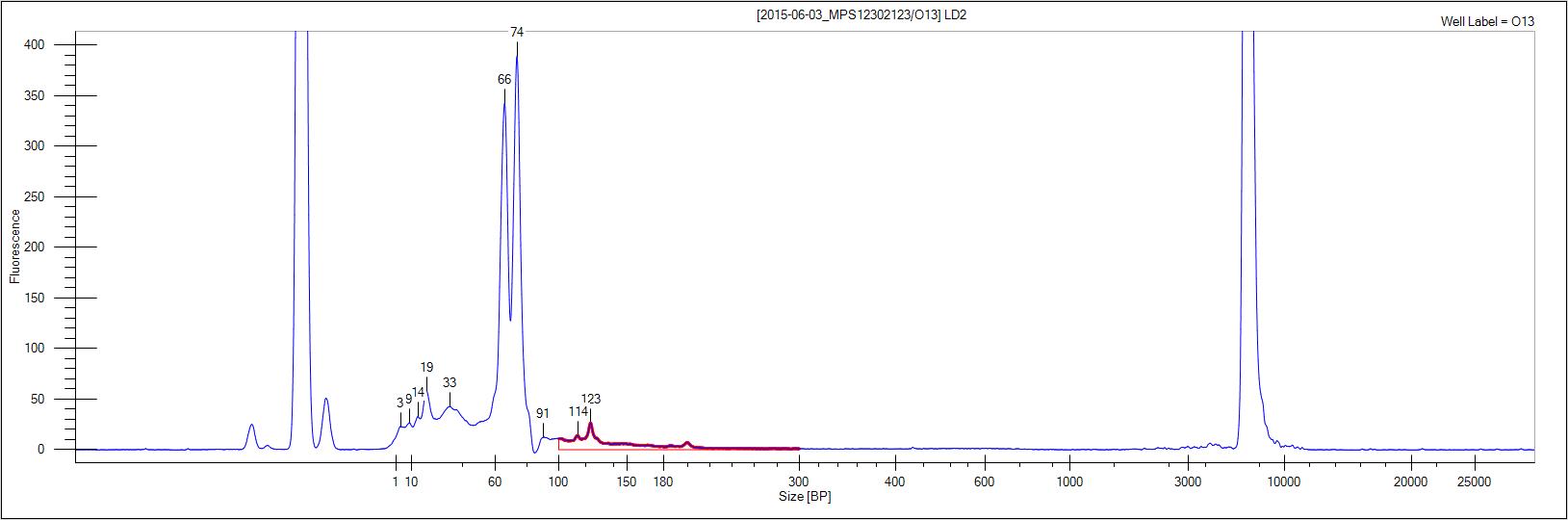

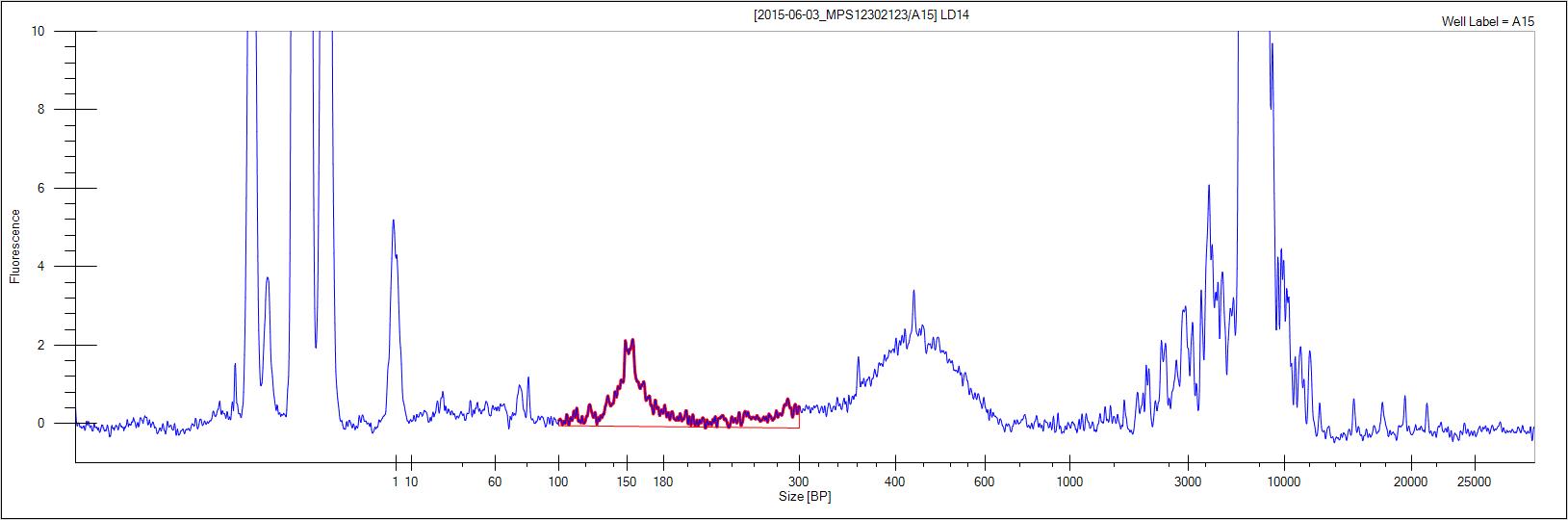
LD2

LD14


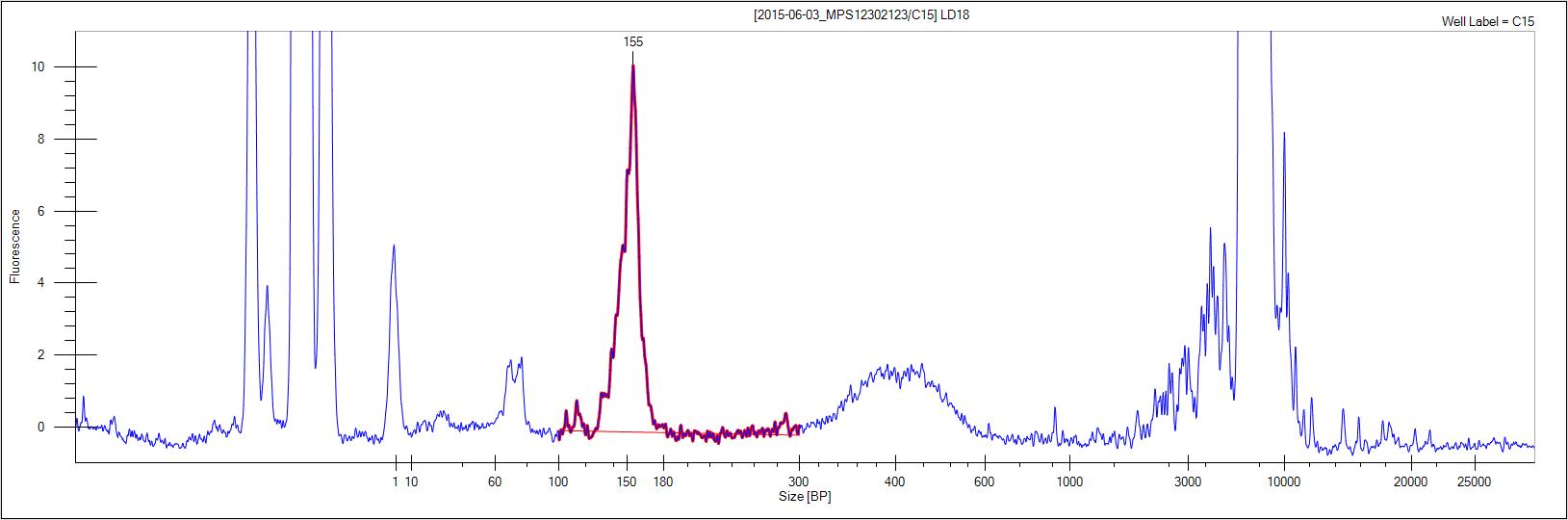
LD18

Supplement: S3 Fig — Three different samples, taken from three different times, were processed by adapter addition and assessed by electrophoresis. The expected size of miRNAs after adapter ligation is ~150 nucleotides. (DOCX) [file pone.0168817.s003.docx]

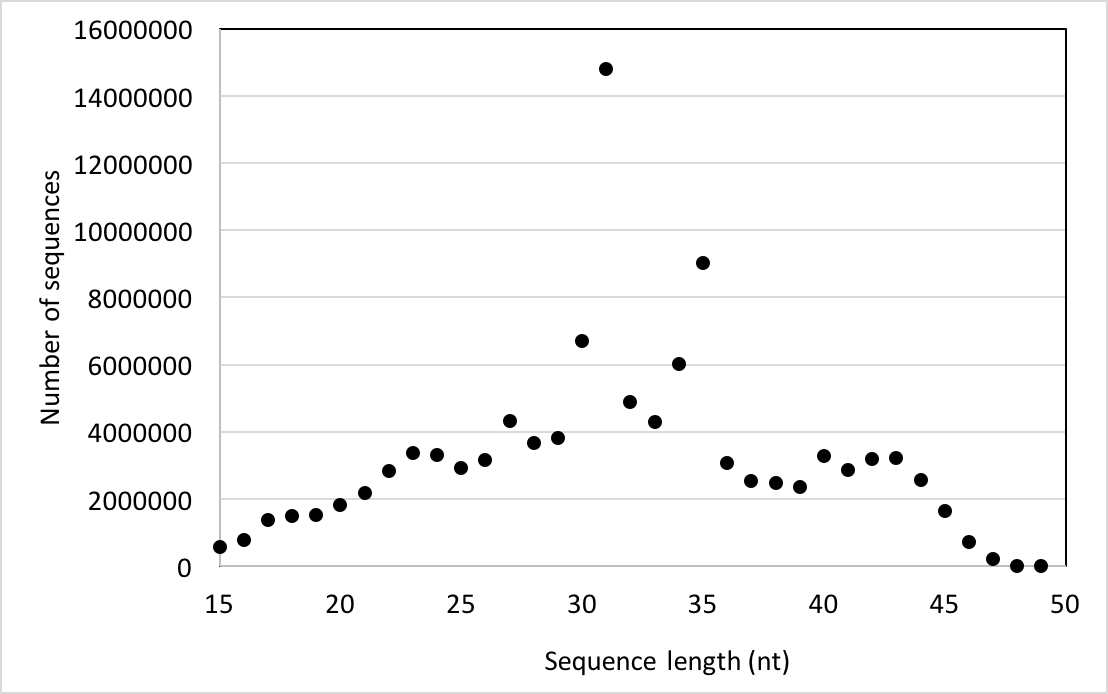

Supplement: S4 Fig — The number of sequences is reported as a function of the sequence length for the 4.9 million small RNAs sequenced. (PNG) [file pone.0168817.s004.png]

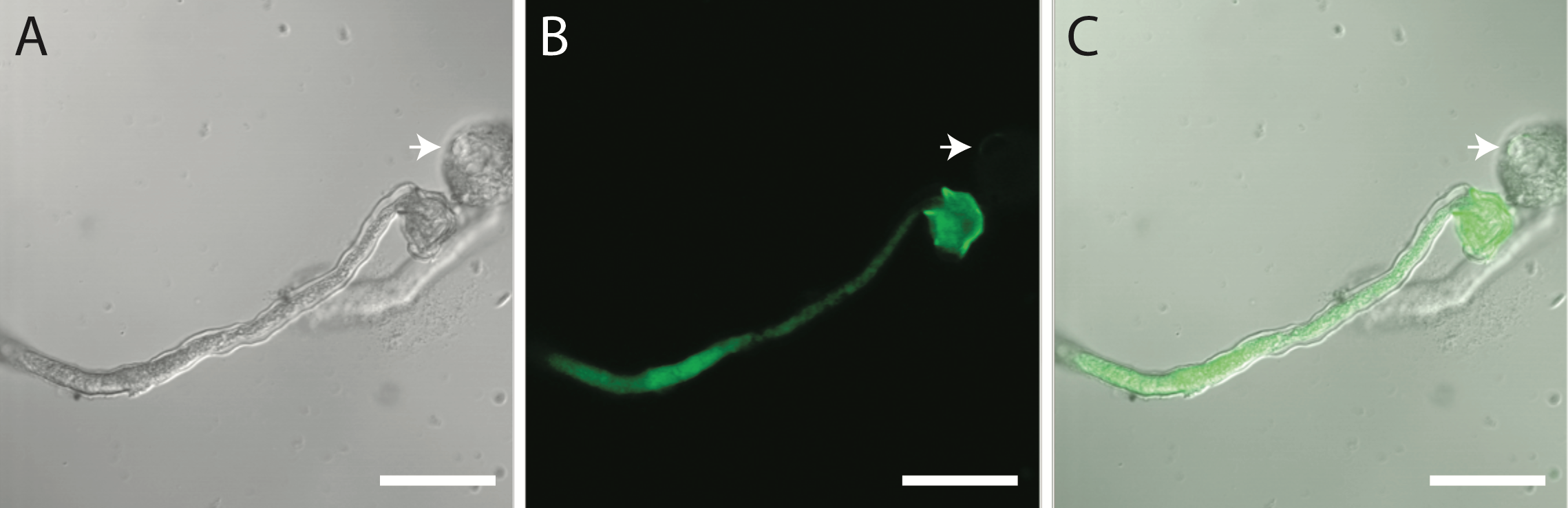

Supplement: S5 Fig — Untransformed pollen has no background GFP fluorescence (arrow). (PNG) [file pone.0168817.s005.png]
