# Supplementary material for: miRNAs Do Not Regulate Circadian Protein Synthesis in the Dinoflagellate Lingulodinium polyedrum
Source: PLoS One. 2017 Jan 19;12(1):e0168817. doi: 10.1371/journal.pone.0168817 (PMC5245829; doi:10.1371/journal.pone.0168817)
Supplement: S1 Table — (PDF) [file pone.0168817.s006.pdf]

S1 Table. The hundred most abundant read sequences map to 28 different transcripts.

Accession	ID (expect value)	FWD Reads	REV reads	Characteristics
GABP01006383	psbC (0)	70080	0	
GABP01022496	atpA	48345	0	
GABP01017680	5S rRNA	46163	0	
GABP01019671	Hypothetical (e^{-35})	20527	0	
GABP01019002	atpB (0)	11605	0	
JO737911	Hypothetical (e^{-35})	10507	0	
GABP01073717	Acyl-CoA thioesterase (0)	9078	0	
GABP01023073	Hypothetical (e^{-18})	3388	0	
GABP01059161	Hypothetical (e^{-17})	2679	0	
GABP01011775	No Significant Hit	2040	0	
GABP01091659	Hypothetical (e^{-151})	1602	0	
GABP01074785	Phosphoglycerate mutase (e^{-109})	1456	0	
GABP01033098	PSII biogenesis protein Psb29 (e^{-29})	1369	0	
GABP01006382	Adenosylhomocysteinase (0)	1339	0	
GABP01080054	No Significant Hit	585	0	
GABP01071941	No Significant Hit	503	0	
GABP01005654	No Significant Hit	163563	1	
GABP01027115	No Significant Hit	12337	1	5 bases differ (2 at 3' end)
GABP01036144	No Significant Hit	126694	2	4 bases differ at 3' end
GABP01112054	Hypothetical (e^{-17})	4084	2	
GABP01020900	No Significant Hit	34793	3	2 bases differ (1 at 3' end)
GABP01078305	p43 (0)	13419	4	7 bases differ at 3' end
GABP01063483	No Significant Hit	14230	7	1-3 bases differ
GABP01058248	No Significant Hit	9410	10	0-6 bases differ
GABP01063174	No Significant Hit	4754	12	4 bases differ at 5' end
GABP01055574	p43 (0)	23817	25	2-6 bases differ
GABP01106675	Glycosyltransferase family 62 (e^{-43})	2861	61	5 bases differ at 3' end
GABP01080345	cox1 (0)	9215	2535	
