# Supplementary material for: miRNAs Do Not Regulate Circadian Protein Synthesis in the Dinoflagellate Lingulodinium polyedrum
Source: PLoS One. 2017 Jan 19;12(1):e0168817. doi: 10.1371/journal.pone.0168817 (PMC5245829; doi:10.1371/journal.pone.0168817)
Supplement: S2 Table — (PDF) [file pone.0168817.s007.pdf]

S2 Table. Predicted miRNA targets.

Duplicate miRNAs and targets are marked in rose.

miRNA_Acc.	Target_Acc.	Expecta	UPE	miRNA_start	miRNA_end	Target_start	Target_end	miRNA_aligned_fragment	Target_aligned_fragment	Inhibition	miRNA orien	Best Target Blast hit
21882-9	JO692627.1	0	16.967	1	20	500	519	UAGAAGAGCUCCUGAUAAGA	UCUUAUCAGGAGCUCUUCUA	Cleavage	antisense	Cytochrome oxidase
10448-16	JO692661.1	0	16.46	1	23	529	551	CAGUUUCCUUCUUGAUUCACGAU	AUCGUGAAUCAAGAAGGAAACUG	Cleavage	sense	Cytochrome oxidase
129572-5	JO692661.1	0	19.155	1	22	327	348	AAGAAUCCCGGACUUUCCAGAU	AUCUGGAAAGUCCGGGAUUCUU	Cleavage		
3435-49	JO692661.1	0	13.768	1	22	529	550	AGUUUCCUUCUUGAUUCACGAU	AUCGUGAAUCAAGAAGGAAACU	Cleavage		
6787-25	JO692661.1	0	15.597	1	22	510	531	GAUUUCUUAGGCGUUGGUCAGC	GCUGACCAACGCCUAAGAAAUC	Cleavage		
6102-27	JO697264.1	2	23.744	1	20	240	259	CGUCCUCGUCGUCCUCGUCG	CGACGAGCACGAUGAGGGCG	Cleavage	sense	NADH oxidase
11330-15	JO697573.1	0	19.531	1	22	4	25	CAGCACCCAAGCAGCACCCAAG	CUUGGGUGCUGCUUGGGUGCUG	Cleavage	antisense	Hypothetical
11330-15	JO697573.1	0.5	23.457	1	22	247	268	CAGCACCCAAGCAGCACCCAAG	CUUGGGUGUUGCUUGGGUGCUG	Cleavage		
11330-15	JO697573.1	1	22.953	1	22	137	158	CAGCACCCAAGCAGCACCCAAG	CUUGGGUGCUGCUUUGGUGCUG	Cleavage		
6102-27	JO699018.1	2	21.79	1	20	1003	1022	CGUCCUCGUCGUCCUCGUCG	CGACGACAACGACGAGGACG	Cleavage	antisense	p43
107948-5	JO702086.1	2	24.892	1	20	3584	3603	AUGUGCGCAUUGAGCUCGGC	GCCGAGCGCAACGCGCACAU	Translation		NA
120862-5	JO702086.1	2	24.892	1	21	3583	3603	AUGUGCGCAUUGAGCUCGGCC	GGCCGAGCGCAACGCGCACAU	Translation		
6102-27	JO702791.1	1.5	24.168	1	20	1569	1588	CGUCCUCGUCGUCCUCGUCG	CGACGAGGACGAGGAGGGCG	Cleavage	antisense	DNAj-like protein
6102-27	JO703428.1	2	22.901	1	20	129	148	CGUCCUCGUCGUCCUCGUCG	CGACGAGGACGACGAUGGCG	Cleavage		NA
6102-27	JO703895.1	2	21.364	1	20	4198	4217	CGUCCUCGUCGUCCUCGUCG	CGAGGAGGACGAGGAGGACG	Cleavage	antisense	ABC transporter
6102-27	JO704271.1	2	22.659	1	20	203	222	CGUCCUCGUCGUCCUCGUCG	CGACGAGGAGGGCGAGGGCG	Translation	antisense	DNA topoisomerase
6102-27	JO704463.1	1.5	16.016	1	20	1180	1199	CGUCCUCGUCGUCCUCGUCG	CGAUGAGGAGGACGAGGACG	Translation	antisense	RNA binding protein 28
6102-27	JO705804.1	0.5	19.534	1	20	640	659	CGUCCUCGUCGUCCUCGUCG	UGACGAGGACGACGAGGACG	Cleavage	antisense	SRP alpha
6102-27	JO706792.1	1.5	21.761	1	20	289	308	CGUCCUCGUCGUCCUCGUCG	CGACGAGUACGGCGAGGACG	Cleavage	antisense	DNAj homologue
6102-27	JO708411.1	2	21.466	1	20	811	830	CGUCCUCGUCGUCCUCGUCG	CGACGACGACGACGAGGACU	Cleavage	antisense	Thylacoid membrane protein TERC
6102-27	JO708578.1	1.5	24.695	1	20	1430	1449	CGUCCUCGUCGUCCUCGUCG	CGACGAGGAGGAUGAGGACG	Translation	antisense	Splicing factor 3B subunit 2
8643-19	JO710260.1	2	22.412	1	21	239	259	CAAUCUUAUCAGGAGCUCUUC	GAAGAGCUCUUGAAGAGAUUG	Cleavage	sense	Unnamed protein
6102-27	JO711265.1	1.5	21.64	1	20	513	532	CGUCCUCGUCGUCCUCGUCG	CGACGACGACGACGAGGAUG	Cleavage		NA
6102-27	JO711474.1	1.5	18.605	1	20	38	57	CGUCCUCGUCGUCCUCGUCG	CGACGAGGAUUACGAGGACG	Translation	antisense	LicD family protein
6102-27	JO712256.1	1	24.3	1	20	780	799	CGUCCUCGUCGUCCUCGUCG	GGACGAGGACGACGAGGACG	Cleavage	antisense	Protein phosphatase 2C
6102-27	JO713144.1	2	17.71	1	20	1311	1330	CGUCCUCGUCGUCCUCGUCG	CUACGAGGACGACGAGGACU	Cleavage	antisense	Sodium/calcium exchanger
6102-27	JO713261.1	2	9.977	1	20	1477	1496	CGUCCUCGUCGUCCUCGUCG	CGACGACGACGACGAGGACA	Cleavage	antisense	Hypothetical
6102-27	JO713977.1	2	15.727	1	20	1676	1695	CGUCCUCGUCGUCCUCGUCG	CGACGAGGAUGACGACGACG	Cleavage	antisense	Protein phosphatase
11330-15	JO715962.1	0	21.595	1	22	301	322	CAGCACCCAAGCAGCACCCAAG	CUUGGGUGCUGCUUGGGUGCUG	Cleavage		NA
11330-15	JO715962.1	1	23.185	1	22	266	287	CAGCACCCAAGCAGCACCCAAG	UUUGGGUGCUGCUUGGGUGCUA	Cleavage		
6102-27	JO716066.1	1.5	18.331	1	20	587	606	CGUCCUCGUCGUCCUCGUCG	CGAUGACGACGACGAGGACG	Cleavage		NA
6102-27	JO719969.1	2	24.444	1	20	1094	1113	CGUCCUCGUCGUCCUCGUCG	CGACGACGACGAGGAGGACG	Cleavage	antisense	RNA recognition family protein
6102-27	JO721329.1	2	22.223	1	20	920	939	CGUCCUCGUCGUCCUCGUCG	CAACGAGGACAACGAGGACG	Translation	antisense	Hypothetical
6102-27	JO723141.1	1.5	23.053	1	20	825	844	CGUCCUCGUCGUCCUCGUCG	CGACGAGUAUGACGAGGACG	Cleavage		NA
6102-27	JO724888.1	1.5	22.736	1	20	209	228	CGUCCUCGUCGUCCUCGUCG	CGACGAGGACGACGAGGAGG	Cleavage	antisense	ABC transporter
6102-27	JO725102.1	2	21.761	1	20	1190	1209	CGUCCUCGUCGUCCUCGUCG	CGCCGAGGACGAGGAGGACG	Cleavage	antisense	Hypothetical
6102-27	JO725781.1	1.5	18.339	1	20	565	584	CGUCCUCGUCGUCCUCGUCG	CGACGAGGAAGAUGAGGACG	Translation		NA
6102-27	JO727049.1	1.5	19.018	1	20	54	73	CGUCCUCGUCGUCCUCGUCG	CGACGAGGAGGGCGAGGACG	Translation		NA
40456-7	JO727816.1	2	16.227	1	20	598	617	CGCCGGACGCGGCGGAGGGG	CUCCUCCGCUGCCUCCGGCG	Cleavage		NA
6102-27	JO727941.1	2	21.125	1	20	924	943	CGUCCUCGUCGUCCUCGUCG	GGAGGAGGACGACGAGGACG	Cleavage	antisense	Hypothetical
6102-27	JO733549.1	1	15.306	1	20	679	698	CGUCCUCGUCGUCCUCGUCG	CGACGACGACGACGAGGACG	Cleavage		NA
6102-27	JO733913.1	2	23.313	1	20	1548	1567	CGUCCUCGUCGUCCUCGUCG	GGACGAGGACAACGAGGACG	Translation		NA
6102-27	JO735152.1	2	18.186	1	20	39	58	CGUCCUCGUCGUCCUCGUCG	UGACGAUGACGACGAGGAUG	Cleavage	sense	ERD1, putative
6102-27	JO735863.1	2	24.411	1	20	762	781	CGUCCUCGUCGUCCUCGUCG	CGCCGAGGACGCCGAGGACG	Translation	antisense	Ricin
6102-27	JO735942.1	2	23.641	1	20	153	172	CGUCCUCGUCGUCCUCGUCG	CGACGAGGACUACGAGGGUG	Translation		NA
6102-27	JO738653.1	2	20.701	1	20	1319	1338	CGUCCUCGUCGUCCUCGUCG	CGACGACGAGGACGAGGACG	Translation		NA
6102-27	JO738742.1	1.5	24.805	1	20	190	209	CGUCCUCGUCGUCCUCGUCG	CGGCGAGGACGAAGAGGACG	Cleavage		NA
6102-27	JO738779.1	2	21.584	1	20	371	390	CGUCCUCGUCGUCCUCGUCG	CAGCGAGGAUGACGAGGACG	Cleavage	antisense	GTP-binding protein
6102-27	JO739509.1	2	12.429	1	20	417	436	CGUCCUCGUCGUCCUCGUCG	UGGCGAGGACGAGGAGGACG	Cleavage	sense	Hypothetical
6102-27	JO741622.1	2	15.223	1	20	424	443	CGUCCUCGUCGUCCUCGUCG	CGACGACGACGACGAGGACU	Cleavage		NA
6102-27	JO746937.1	2	18.059	1	20	651	670	CGUCCUCGUCGUCCUCGUCG	UGACGAGGACGACGAGGAGG	Cleavage		NA
6102-27	JO747914.1	2	22.995	1	20	1794	1813	CGUCCUCGUCGUCCUCGUCG	CGACGAGGAUGACGAGGAGG	Cleavage	antisense	Protein kinase
6102-27	JO750816.1	2	14.821	1	20	141	160	CGUCCUCGUCGUCCUCGUCG	CGACGAGGGCGACGAGGGCC	Cleavage		NA

11330-15	JO758852.1	1	16.163	1	21	1	21	CAGCACCCAAGCAGCACCCAA	UUGGGUGCUGGUUGGGUGCUG	Translation		na
11330-15	JO758852.1	1	24.867	1	21	136	156	CAGCACCCAAGCAGCACCCAA	UUGGGUGCUGGUUGGGUGCUG	Translation		
11330-15	JO758852.1	1.5	17.12	1	21	23	43	CAGCACCCAAGCAGCACCCAA	UUGGGUGCUGGUUGGGUGUUG	Translation		
11330-15	JO758852.1	1.5	19.711	1	21	79	99	CAGCACCCAAGCAGCACCCAA	UUGGGUGCUGGUUGGGUGUUG	Translation		
6102-27	JO759126.1	2	16.513	1	20	116	135	CGUCCUCGUCGUCCUCGUCG	AGACGAGGACGAGGAGGACG	Cleavage		NA
6102-27	JO761233.1	1.5	22.28	1	20	1640	1659	CGUCCUCGUCGUCCUCGUCG	CGACGAGGACGACGAUGACG	Cleavage	antisense	Karyopherin
6102-27	JO766993.1	1.5	20.965	1	20	323	342	CGUCCUCGUCGUCCUCGUCG	UGAUGAGGACGACGAGGAUG	Cleavage	antisense	Cytochrome oxidase
10890-16	JO767378.1	0	15.605	1	22	71	92	CCCGGAGACAGAAAGUGAGAGA	UCUCUCACUUUCUGUCUCCGGG	Cleavage		NA
7324-23	JO767378.1	0	18.849	1	22	161	182	UCGUGAAUCAAGAAGGAAACUG	CAGUUUCCUUCUUGAUUCACGA	Cleavage		
33181-7	JO767419.1	2	12.945	1	23	19	41	CUGGGGUGAUGCUUGGGUGCUGC	GCAGCGGCCAAGCAUCAUCCCAG	Cleavage		NA
4933-34	JO767419.1	0	12.942	1	24	40	63	CUUGGGUGCUGCUUGGGUGCUGCU	AGCAGCACCCAAGCAGCACCCAAG	Cleavage		
4933-34	JO767419.1	0	8.616	1	24	316	339	CUUGGGUGCUGCUUGGGUGCUGCU	AGCAGCACCCAAGCAGCACCCAAG	Cleavage		
4933-34	JO767419.1	1	11.23	1	24	143	166	CUUGGGUGCUGCUUGGGUGCUGCU	AGCAGCACCCAAGCAGCGCCCAMG	Cleavage		
4933-34	JO767419.1	1.5	13.364	1	24	65	88	CUUGGGUGCUGCUUGGGUGCUGCU	AGCAGCACCCAAGCAGCACCCACG	Cleavage		
5105-33	JO767419.1	0	14.274	1	23	40	62	UUGGGUGCUGCUUGGGUGCUGCU	AGCAGCACCCAAGCAGCACCCAA	Cleavage		
5105-33	JO767419.1	0	8.62	1	23	316	338	UUGGGUGCUGCUUGGGUGCUGCU	AGCAGCACCCAAGCAGCACCCAA	Cleavage		
5105-33	JO767419.1	1	13.364	1	23	65	87	UUGGGUGCUGCUUGGGUGCUGCU	AGCAGCACCCAAGCAGCACCCAC	Cleavage		
5105-33	JO767419.1	1	11.326	1	23	143	165	UUGGGUGCUGCUUGGGUGCUGCU	AGCAGCACCCAAGCAGCGCCCAM	Cleavage		
5105-33	JO767419.1	1.5	12.817	1	23	232	254	UUGGGUGCUGCUUGGGUGCUGCU	AGCAGCACCCAAGCAGCGCCCAC	Cleavage		
51061-6	JO767419.1	1.5	12.942	1	24	40	63	CUUGGGUGCUGUUUAGGUGCUGCU	AGCAGCACCCAAGCAGCACCCAAG	Cleavage		
51061-6	JO767419.1	1.5	8.616	1	24	316	339	CUUGGGUGCUGUUUAGGUGCUGCU	AGCAGCACCCAAGCAGCACCCAAG	Cleavage		
6755-25	JO767419.1	1.5	14.274	1	23	40	62	UUGGAUGCUGCUUGGGUGCUGCU	AGCAGCACCCAAGCAGCACCCAA	Cleavage		
6755-25	JO767419.1	1.5	8.62	1	23	316	338	UUGGAUGCUGCUUGGGUGCUGCU	AGCAGCACCCAAGCAGCACCCAA	Cleavage		
78494-5	JO767419.1	1.5	14.274	1	23	40	62	UUGGGGGCUGCUUGGGUGCUGCU	AGCAGCACCCAAGCAGCACCCAA	Cleavage		
78494-5	JO767419.1	1.5	8.62	1	23	316	338	UUGGGGGCUGCUUGGGUGCUGCU	AGCAGCACCCAAGCAGCACCCAA	Cleavage		
12389-14	JO767429.1	0	8.974	1	21	177	197	UAAUGAACGUCAGGCACCUGC	GCAGGUGCCUGACGUUCAUUA	Cleavage		NA
